# Amyand's Hernia: Perforated Appendix in an Incarcerated Inguinal Hernia

**DOI:** 10.7759/cureus.7622

**Published:** 2020-04-10

**Authors:** Anupam K Gupta, Oscar A Vazquez, James El Haddi, Michael Dedwylder, Jose F Yeguez

**Affiliations:** 1 Surgery, Charles E. Schmidt College of Medicine, Florida Atlantic University, Boca Raton, USA

**Keywords:** surgery, amyand's hernia

## Abstract

Amyand’s hernia is an unusual condition characterized by the presence of a normal or inflamed appendix located within an inguinal hernia. We present a rare situation wherein a 56-year-old male patient presented with an incarcerated inflamed appendix in a right inguinal hernia. He was emergently taken to the operating room, with diagnostic laparoscopy changed to open, due to incarcerated cecum and terminal ileum. The incarcerated segment had to be resected with primary anastomosis. The inflamed and purulent contents were washed out, and the hernia defect was left unrepaired due to the presence of abscess in the inguinal canal.

## Introduction

Inguinal hernias account for 75% of abdominal wall hernias, with a lifetime risk of 27% in men and 3% in women [1]. Incarceration of the appendix within an inguinal hernia is termed Amyand’s hernia, named after Dr. Claudius Amyand, who performed the first appendectomy where the appendix happened to be located in the inguinal canal. An Amyand’s hernia can become inflamed, infected, or perforated, or be incarcerated and completely healthy. Furthermore, it is difficult to diagnose preoperatively and is most often an intraoperative finding [2]. A classification system for Amyand’s hernia has been proposed to guide treatment. Type I hernia has a normal appendix in an inguinal hernia. Types II to IV have acute appendicitis within an inguinal hernia sac. Type II has an inflamed non-perforated appendix. Type III has a perforated appendix and type IV is complicated with intra-abdominal pathology. Types I and II are managed with reduction and mesh repair or appendectomy and mesh repair. In types III and IV, hernioplasty may be contraindicated due to spread of sepsis or extensive damage [3].

## Case presentation

A 56-year-old male presented to the emergency department with symptoms of acute right groin pain associated with a bulge. The patient’s pain was severe and was 10/10 on the pain scale which was not relieved with oral pain medication at home. Symptoms over the last few days included subjective fevers, chills, and cough for which the patient was currently on day 2 of an ongoing, outpatient course of azithromycin and oral methylprednisolone for pneumonia. The patient’s past medical history was significant for non-insulin-dependent diabetes mellitus for which he was non-compliant with his home regimen. On arrival to the emergency room, the patient had a temperature of 37.9°C oral, a heart rate of 101 beats per minute (sinus tachycardia), a blood pressure of 140/74 mmHg, and an oxygen saturation of 95% on room air. On clinical examination, the patient had an incarcerated right inguinal hernia which was very tender to touch. Routine blood work and a basic chemistry profile done at time of arrival were significant for leukocytosis to 21.1x10^3^/µL, and the rest of the labs were largely unremarkable.

**Figure 1:**
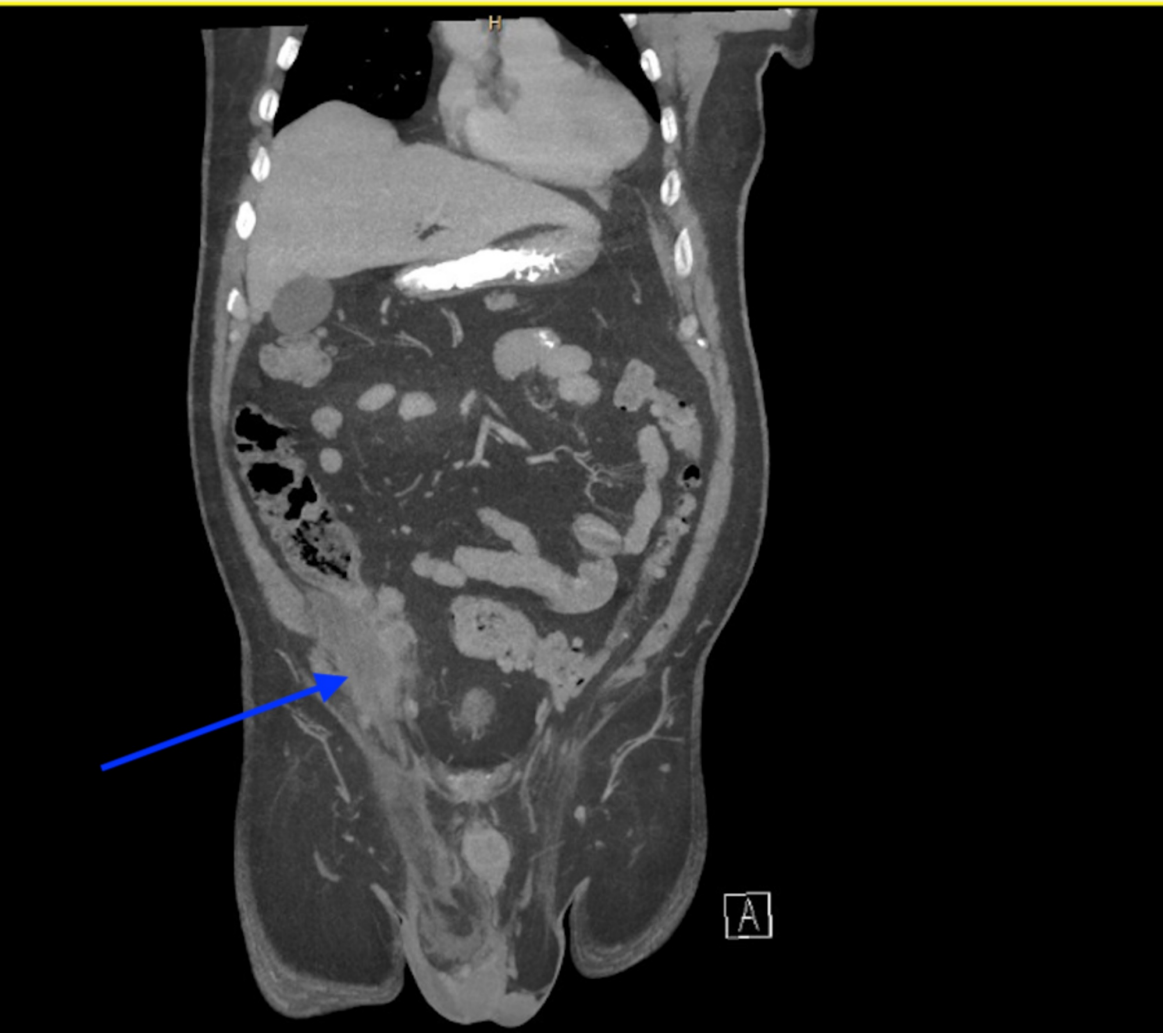
Right groin hernia with inflammatory changes around the contents.

**Figure 2:**
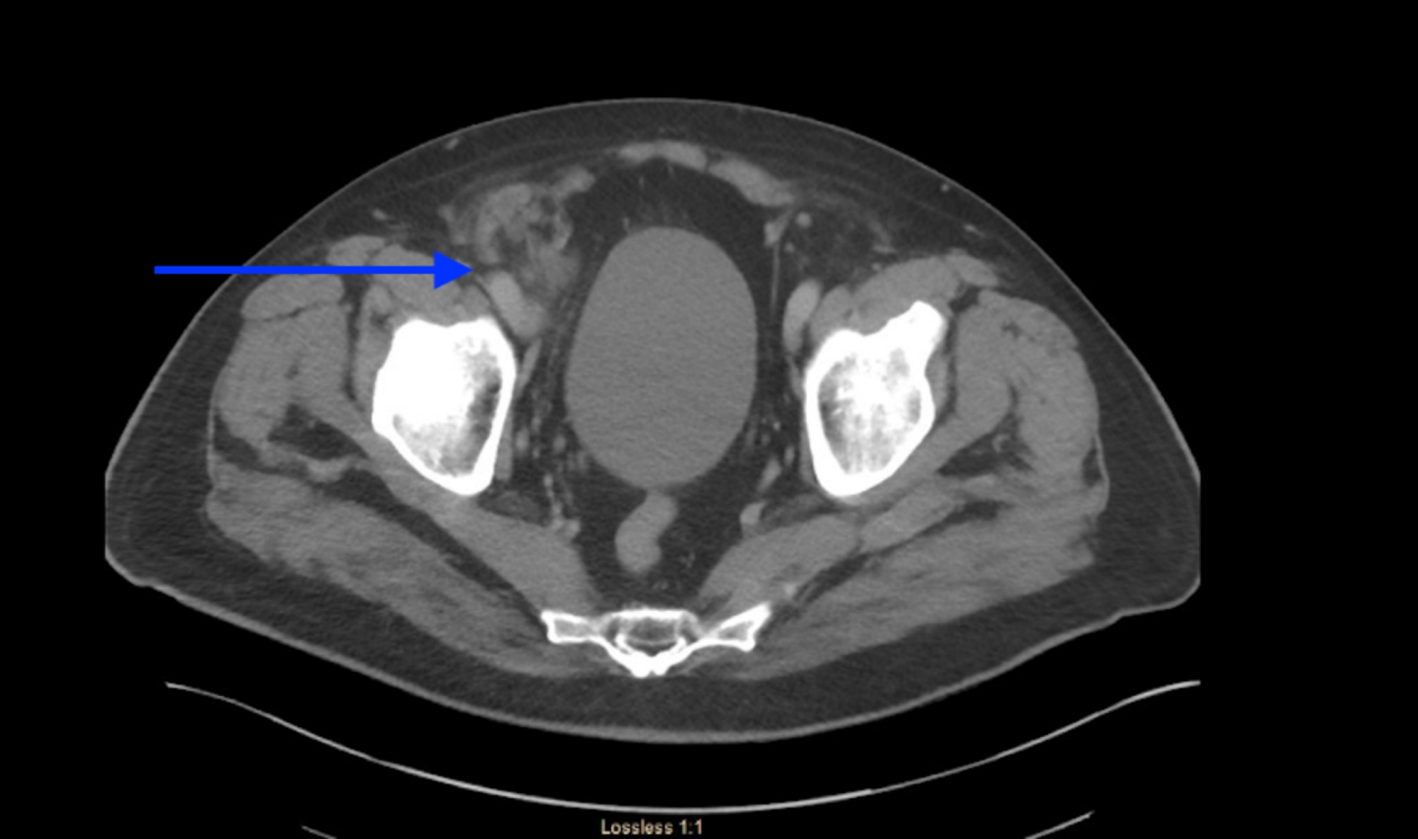
Appendix herniating into right groin hernia with inflammatory changes surrounding it.

**Figure 3:**
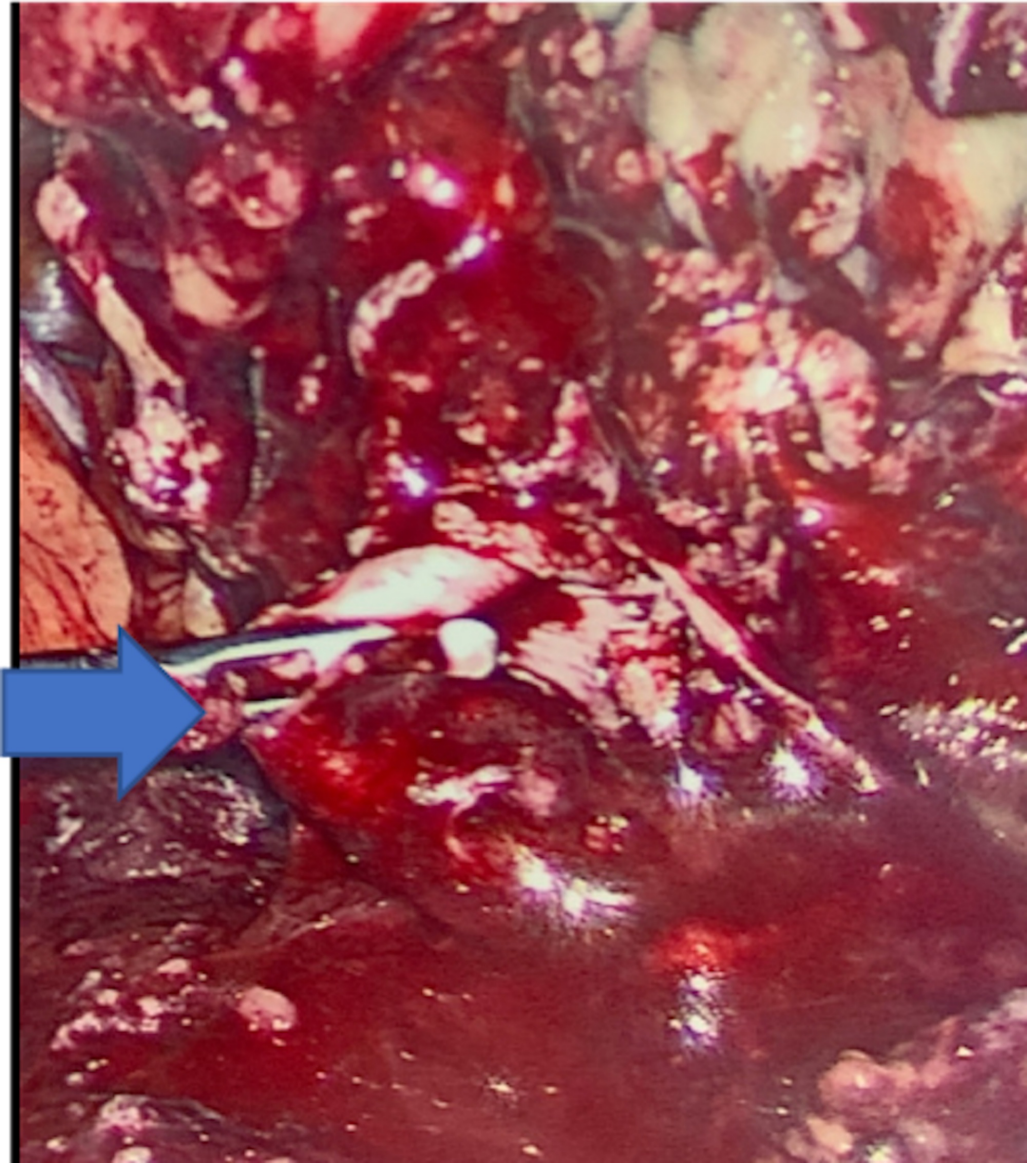
Inflamed appendix and surrounding inflammation entering the right inguinal hernia orifice.

**Figure 4:**
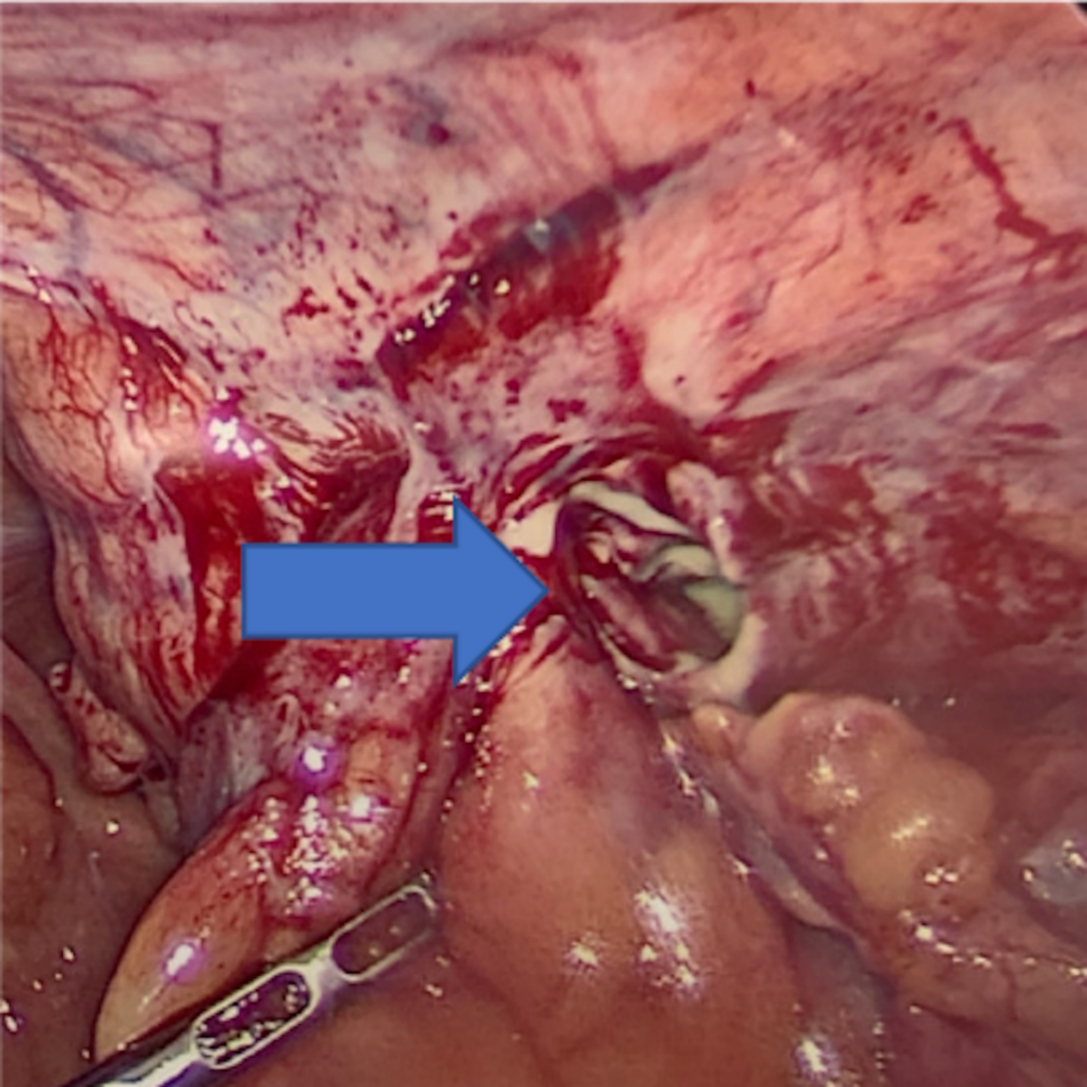
After reduction of contents showing purulent material in the right inguinal hernia orifice.

Computed tomography (CT) of abdomen and pelvis showed an inflamed appendix herniating into a right inguinal hernia. The appendix had evidence of extensive inflammatory changes surrounding it (Figures 1, 2). The patient was emergently taken to the operating room for diagnostic laparoscopy. During laparoscopy it was observed that terminal ileum, perforated appendix, and cecum were incarcerated in the hernia sac with intense inflammation surrounding it (Figures 3, 4). In attempt to reduce the contents, there was extensive drainage of purulent material consistent with a contained perforation into the inguinal canal. A serosal tear was noted on the ileum and cecum which prompted the decision to perform an exploratory laparotomy via a midline incision. In view of these extensive inflammatory changes and serosal tears, the patient had to undergo resection of the terminal ileum of approximately 5 cm, cecum, appendix, and right colon. A side-to-side functional end-to-end ileotransverse anastomosis using the Endo-GIA 60-mm blue load stapler (Ethicon, Somerville, NJ) was performed at the site with healthy bowel loops without any inflammatory changes. A 10-French flat Jackson-Pratt drain (Cardinal Health, Dublin, OH) was placed in the right iliac fossa into the hernia canal to drain the purulent material after thorough saline irrigation. The abdominal fascia was closed primarily using running PDS sutures. Postoperative course was complicated by the development of abscess in the right groin which needed drainage on postoperative day 4 via scrotal incision. The patient was discharged to home on postoperative day 7 with one-week course of cephalosporin for Escherichia coli growth in wound.

## Discussion

The incidence of Amyand’s hernia ranges in the literature from 0.19% to 1.7% of reported hernia cases. Amyand’s hernia is three times more likely to be diagnosed in children than in adults due to the patency of the processus vaginalis in the pediatric population [4]. The incidence of appendicitis within an inguinal hernia is even more rare with an estimated rate at 0.07%-0.13% [5]. In addition, the incidence of perforated appendix incarcerated within an inguinal hernia is rare as well, at 0.1% of all cases of appendicitis [6]. Mortality of Amyand’s hernia has been reported to range from 14% to 30% most likely caused by sepsis; however, another study found a mortality rate of only 5.5% with early appropriate treatment and good postoperative care [2,5].

Common complaints of this diagnosis include sudden onset epigastric or periumbilical pain with localized tenderness in the right lower quadrant, combined with a tender irreducible mass in the inguinal or inguinoscrotal region [7]. This presentation makes diagnosis of Amyand’s hernia difficult as it gives the clinical impression of a strangulated hernia [8]. Abdominal exam, physical signs, lab results, and imaging are usually not helpful in the differential diagnosis [9,10]. CT is the most commonly used imaging modality for evaluation of acute abdomen and abdominal hernias. However, inguinal hernias are typically diagnosed clinically [11]. The lack of specific presenting signs and symptoms in Amyand’s hernia, even when complicated, means that imaging is commonly not ordered if a simpler diagnosis can be made clinically. In the instances when imaging is ordered, it is usually for the purpose of ruling out a more serious pathology [12]. The most common choice of treatment for Amyand’s hernia is appendectomy via herniotomy with primary hernia repair [13,14]. Lower midline laparotomy is recommended for cases of suspected perforation or pelvic abscess. Hernia repair is usually completed during primary surgery but may need to be delayed due to complications and inflammation [3,15].

## Conclusions

Amyand’s hernia is an unusual hernia with appendix as one of its contents. A perforated appendix in the groin hernia is rare. This is an emergent condition needing source control over infection to control sepsis. In view of intense inflammation, surgical repair of the hernia is often not feasible and needs to be staged at a later date.
